# Just “Like Coffee” or Neuroenhancement by Stimulants?

**DOI:** 10.3389/fpubh.2021.640154

**Published:** 2021-06-07

**Authors:** Andreas G. Franke, Gabriele Koller, Daniela Krause, Lisa Proebstl, Felicia Kamp, Oliver Pogarell, Tarek Jebrini, Kirsi Manz, Agnieszka I. Chrobok, Michael Soyka

**Affiliations:** ^1^Hochschule der Bundesagentur für Arbeit/University of Applied Labour Studies, Mannheim, Germany; ^2^Department of Psychiatry and Psychotherapy, Hospital of the Ludwig Maximilian University, Munich, Germany; ^3^Institute for Medical Information Processing, Biometry, and Epidemiology, Ludwig Maximilian University, Munich, Germany

**Keywords:** caffeine, coffee, energy drinks, neuroenhancement, misuse

## Abstract

**Introduction:** Pharmacological neuroenhancement (PN) is a topic of increasing importance and prevalence among students. However, there is a lack of differentiating PN substances, according to their psychoactive effects. In particular, there is a lack of data about PN by caffeinated drinks, even if coffee is a common and broadly used Neuroenhancer because of its cognitively enhancing effects regarding wakefulness, alertness and concentration.

**Materials and Methods:** A web-survey was developed for German students and alumni about the non-medical use of caffeine for PN contained questions about coffee, caffeinated drinks and energy drinks, caffeine pills and methylxanthine tea regarding frequency and further contextual factors.

**Results:** Six hundred and eighty-three participants completed the survey. Nearly all participants knew about PN (97.7%). 88.1% admitted using some over-the-counter substances. For PN purposes, coffee was used by 72.9% followed by energy drinks (68.2%) and cola drinks (62.4%). Methylxanthine containing tea was used for PN purposes, too (black tea 52.3%, green tea 51.7%). 1.8% admitted using illegal substances or prescription drugs, too.

**Discussion:** Using legal methylxanthine containing drinks for PN seems to be extremely common with coffee and energy drinks being the preferred substances, while illegal and prescription drugs are only minimally used. Further studies should investigate the awareness of methylxanthine containing drinks as well as its character to be a flavoring drink or a neuroenhancer.

## Introduction

The article “Poll results: Look who is doping” is one of the most cited articles in the field of pharmacological neuroenhancement (PN) (1). In 2008, Nature ran this online poll among their readers to study the frequency and reasons regarding the use of psychoactive substances to enhance cognitive performance. Until today, this article can be considered as “conversation starter.” However, meanwhile there are much more studies examining the phenomenon of using substances to increase cognition deeper and much more systematic.

The term “smart drugs” is used for this group of drugs, as well as other synonyms e.g., brain doping, academic performance enhancement, cognitive enhancement or pharmacological neuroenhancement (PN). PN is mostly defined as the non-medical use of divergent psychoactive substances to increase vigilance, attention, concentration or memory by healthy subjects (2–4).

The above mentioned poll assessed the use of methylphenidate, modafinil and beta blockers for cognitive enhancement. The authors demonstrate that 20% of the 1,400 participants had used at least one of the aforementioned substances to improve their focus, concentration or memory without medical need (1). Meanwhile there are several national and international publications about the use of prescription as well as illicit substances for PN. They show lifetime prevalence rates of 1 up to 20% depending on the substances assessed, the survey methods used and other factors (5–10). However, until today there is a paucity of studies regarding legal over the counter (OTC-) substances such as caffeine for PN and their contextual factors. Although, caffeine—standing for the best well-known representative of the chemical group of methylxanthines (such as caffeine, theobromine, theophylline) (11)—has proved pro-cognitive effects [e.g., (12–16)]. Methylxanthines are legal alternative PN substances compared to prescription and illicit substances (e.g., amphetamines, modafinil, etc.). Regarding this comparison, methylxanthines are frequently preferred by several students based on ethical, legal and medical reasons (17). Beyond that, the use of coffee has to be considered as a cultural habit with the well-known “side effect” of having wake promoting properties—especially when being tired (11).

“Real” side effects such as jitteriness, sleeplessness, stomachache etc. have to be considered when using methylxanthine containing substances. Side effects are listed inter alia in the so called “Arzneimittelfachinformation” of the only caffeine containing tablet in Germany (Coffeinum®). Of course, these possible side effects can harm users but they can be considered as “minor” side effects compared to the side effects of amphetamines, methylphenidate or modafinil [e.g., (18)]. Nevertheless, when deciding to use a PN substance, students make their individual decision regarding the choice of the type of the PN substance in parts based on ethical aspects but mainly dominated by medical and legal aspects (17).

Even if caffeinated substances epidemiologically seem to be well-known for PN [e.g., (7, 19–21)], there is a paucity of studies enabling a deeper understanding of this context. For example, Forlini et al. showed that most students knew about the possibility to use coffee, caffeinated drinks and caffeine tablets for PN; in this study 56% indicated a past use of coffee and 41% a past use of energy drinks for PN purposes (20). Franke et al. showed similar results in 2011 (22). In sum, superficial aspects about caffeine for PN have been studied while systematic data about the use of methylxanthines combined with contextual factors for a deeper understanding are missing.

An older survey on caffeine use in university students showed, lifetime, past-year and past-month prevalence rates for the use of coffee specifically for PN of 53.2, 8.5, and 6.3%, for the use of energy drinks of 39, 10.7, and 6.3%, and for the use of caffeine tablets of 10.5, 3.8, and 0.8% (22). Additionally, a survey study among surgeons revealed lifetime, past-year, past-month, and past-week prevalence rates for coffee specifically for PN of 66.8, 61.9, 56.9, and 50.5% (2). For energy drinks they found prevalence rates of 24.2, 15.4, 9.9, and 6.1% and for caffeine tablets of 12.6, 5.9, 4.7, and 3.8% (2). Both studies showed the use in light of stress, pressure to perform and reduction of fatigue (2, 22). However, these and other studies do not give deeper insights in methylxanthine use for PN such as psychotropic and side effects, the amount of used cups of coffee per day, product names of energy drinks, etc.

The mechanism of action of methylxanthines has been investigated in the past (11) and leads to states of increased cognitive abilities (reduced fatigue, increased wakefulness, concentration, shortened reaction time, etc.) (2, 23, 24). Clinically, pro-cognitive effects of caffeine have been shown to be comparable to effects of stimulants such as amphetamines: Randomized controlled trials showed 600 mg of caffeine to have comparable clinical effects to 20 mg of dextro amphetamine or 400 mg of modafinil in healthy sleep-deprived subjects (at least regarding the restoration of simple psychomotor performance and objective alertness) (13–15). Beyond that, a comparison between coffee and the so called “energy drinks”—having additional ingredients such as taurine—showed that energy drinks have stronger clinical effects regarding cognitive PN domains than coffee (25).

Beyond coffee, methylxanthine containing tea and energy drinks, there are caffeine pills containing different amounts of caffeine. In Germany, Coffeinum® is sold in specialized pharmacy stores, each pill containing 200 mg of caffeine. It is approved for short time reduction of tiredness. This amount of caffeine “per pill” is less than the amount of caffeine usually found in a grande coffee in a coffee shop containing more or less 500–600 mg of caffeine.

The character of caffeine has three different faces: (1) coffee primarily as a flavoring beverage, (2) energy drinks (and other beverages and foods) containing caffeine as a food supplement, and (3) caffeine tablets that have to be regarded as a drug/medication.

Summing up, caffeine in various routes of administration seems to be a widespread PN drug. The present web-based study was designed to improve the current database on knowledge and prevalence rates by further contextual factors such as differentiating methylxanthines, frequency and amount of use and further factors such as effects, side effects as well as a combination with other psychoactive substances.

Therefore, this preliminary study assessed a convenience sample of students, alumni and associated people at a University of Applied Sciences (UAS) in Mecklenburg-Vorpommern, a federal German state, about their use of methylxanthine containing substances and drinks. For a literature comparison and for having a basis about PN drug use, data about the general knowledge of PN and the use of prescription and illicit substances were collected, too. However, mainly this preliminary study wants to open a new chapter of methylxanthines research for PN.

## Materials and Methods

The present study was designed as an online survey using the survey tool “Unipark” and distributed among employees, students and alumni of the University of Applied Sciences (UAS) in Neubrandenburg (NB), in Mecklenburg-Vorpommern, a federal state in north-eastern part of Germany. Announcements for the survey were distributed *via* electronic media: homepage, social media and mailing lists of the above mentioned UAS. The invitations were posted and mailed in June 2016; the survey was opened between July and September 2016.

In total 717 participants participated in the survey. Participants with missing data (*n* = 34; 4.7%) had to be excluded. The remaining 683 participants have been included in the analysis of the survey.

The data was acquired using a self-designed online survey to ensure a high degree of privacy and anonymity for all participants. Before the questionnaire starts, participants were informed about the aim and the content of the questionnaire. A section explaining the emphasis on caffeine use for PN was given in bold letters. The chemically correct term “methylxanthine” was avoided and replaced by the word “caffeine” to make the emphasis clearer for all participants.

PN was defined in the introduction of the questionnaire to be the use of divergent substances (drugs and drinks) with the specific aim to increase cognition (e.g., wakefulness, attention, concentration, memory) without needing these substance(s) as a medication because of an existing disease.

### Description of the Questionnaire

The questionnaire was built as follows: after questions about demographic data (sex, age, professional status: student, employed, student and employee, other), the questionnaire asked for data about knowledge of PN drugs in general to build a basis of PN data comparable to the present literature: having ever heard about those by type of source of knowledge [print media, TV, internet, colleagues, friends/relatives, (other) students], type/class of substance: over-the-counter (OTC) drugs (including caffeine, Ginkgo biloba, etc.), prescription drugs (methylphenidate, e.g., Ritalin®) and illicit drugs (illicit amphetamines e.g., “Speed”); having used substances with the particular intention of PN and frequency (never, during last month, during last year, longer than 1 year ago) of the use of (a) coffee, (b) energy drinks (e.g., Red Bull®), (c) caffeine pills (e.g., Coffeinum®), (d) caffeine drinks (e.g., Coca Cola®), (e) black tea, (f) green tea (g) illicit drugs, and (h) prescription drugs.

The questionnaire then opened a new chapter on the use of methylxanthine containing substances for PN and asked the following questions about aspects of using methylxanthine containing substances: age at first use, motivation/reason for the use, the subjectively observed/felt neuroenhancing effects, side effects, the habit of mixing the energy drink with alcohol, average number of cups of coffee used per day (not for PN purposes). For each energy drink, participants had the possibility to specify the brand name.

A pre-test of the questionnaire was conducted among a dozen of voluntary participants. Based on this pre-test, the questionnaire was adapted for the survey. Especially the wording (methylxanthine → caffeine containing, caffeinated → drink energy drink) was changed to increase understandability for future participants.

### Data Analysis

Data were collected and stored in the Unipark database. Data were extracted as an Excel file and converted to SPSS (Ver 25.0) which was used for statistical calculations. Variables had to be (re-) named/characterized according to the variables of the questionnaire.

Binary univariable logistic regression analyses were used to predict the use of OTC or illicit and/or prescription drugs, respectively. As predictor variables age, gender and professional status were included. For non-normally distributed continuous variables, Mann-Whitney U test was applied to test for differences in the mean between two groups. For two normally distributed variables a *t*-test was performed. The association between categorical variables was assessed by means of Fisher's exact-test. For all analyses we consider a *p*-value of below 0.05 indicating statistically significant effects. Since our study was designed as a first, preliminary explorative study, no *p*-value adjustment for multiple testing was considered.

In the first part of the questionnaire about PN in general, single substance names (e.g., methylphenidate, amphetamine, speed, etc.) were used to simplify the answering process for all participants. Consistent with the focus of the study (methylxanthines for PN), substance names were grouped as follows: Participants who reported using “Speed,” Ritalin®, cocaine, Neurodoron®, ephedrine, amphetamines (including illicit), prescription drugs for insomnia, ecstasy, MDMA, or other illicit substances were categorized as those using illicit or prescription drugs for PN. Prescription and illicit drugs were not specifically distinguished.

### Ethics Statement

The study was performed according to the Declaration of Helsinki (1975, revised 2000). Participants gave informed consent by clicking on a button after reading a short introductory paragraph and by pressing the button “done” at the end of the survey. This procedure as well as the whole study was approved by the responsible ethics committee (Neubrandenburg; Approval No. BB 045/14).

## Results

Most of the participants were female (72.6%), with a mean age of 26.6 years (Table 1). The main group that participated in the survey were students (81.3%). Further participants were recent alumni of the same university and were employees, or built a group of “double-strain” persons (due to their status as students as well as employees) (see Table 1).

**Table 1:** Basic characteristics of the survey participants (*n* = 683).

**Age^a^**	***M* = 26.6**	***SD* = 6.6**
Gender	Male	187 (27.4%)
	Female	496 (72.6%)
Profession	Students and trainees	555 (81.3%)
	Employees	93 (13.6%)
	Double stress^b^	22 (3.2%)
	Others	13 (1.9%)

*Mean (M) and standard deviation (SD), or number and percentage of participants are shown*.

a *Age in years*.

b *Meaning students that are also employed*.

Almost all of the 683 participants (97.7%) had already heard about the possibility of using substances for PN purposes (see Table 2). Knowledge about PN substances came from fellow students (56.5%), TV (56.5%), the internet (54.5%), friends and family (52.0%), and print media (47.0%). Sharing the knowledge about PN with colleagues was reported considerably less often (23.9%).

**Table 2:** General knowledge of pharmacological neuroenhancement according to the type of source where this knowledge came from.

**Have you ever heard about neuroenhancement drugs?**	**Yes**	**Print media**	**TV**	**Internet**	**Colleagues**	**Fellow students**	**Friends and family**
	667 (97.7%)	*n* = 321 (47.0%)	*n* = 386 (56.5%)	*n* = 372 (54.5%)	*n* = 163 (23.9%)	*n* = 386 (56.5%)	*n* = 355 (52.0%)

After the above mentioned general question about participants' knowledge of PN, the questionnaire asked explicitly for the use of methylxanthine containing substances/drinks for PN (see Table 3).

**Table 3:** Use of OTC an illegal/prescription drugs according to the professional status (*n* = 683).

**Professional status**	**OTC drugs**	**Illegal/prescription drugs**
Employees	89.2% (*n* = 83)	1.1% (*n* = 1)
Students	88.1% (*n* = 489)	2% (*n* = 11)
“Double-stress”	95.5% (*n* = 21)	0%
Others	69.2% (*n* = 9)	0%

The vast majority of all participants (88.1%, *n* = 602, lifetime prevalence) admitted to have used some of the methylxanthine containing OTC substances. Only 1.8% of all participants admitted to use illegal or prescription substances (without medical prescription). The use of OTC and/or illegal substances according to the professional status is shown in Table 2. These results suggest that the rates among employed persons and students, when it comes to using OTC substances, are equally high. However, prevalence rates among double stressed (being student as well as employee) participants were higher (95.5%) (see Table 3).

No gender differences between the use of methylxanthine containing OTC substances for PN or the use of illegal or prescription substances for PN could be found (*p* = 0.963). The same applies for the association between professional status and gender (data not shown).

Regarding the frequency (ever, last year, last month) of using methylxanthine containing substances/drinks for PN, the most commonly used substance/drink was coffee (72.9%) followed by energy drinks 68.2% and cola drinks (62.4%). Methylxanthine containing tea was used for PN purposes, too: Fifty two percent of the participants used black tea and 51.7% green tea. Regarding caffeine tablets, lifetime prevalence was 28.7% (for further data see Table 4). Only 1.8% of the participants admitted using illegal substances or substances only available on prescription (data not shown in the table). The most often reported illegal/prescription substances were amphetamine type substances (0.5%). Prevalence rates during “the last 30 days” were considerably higher than the use of “more than 12 months ago” and the use “within the last 12 months” which was applicable for all methylxanthine containing substances/drinks except caffeine pills. The most frequently used PN substance/drink (49.5%) was coffee. For more details, please see Table 4.

**Table 4:** Prevalence rates for the use of legal neuroenhancement substances.

**Substance used for neuroenhancement**	**Never**	**Within the last 30 days**	**Within the last 12 months**	**More than 12 month ago**
Coffee	185 (27.1%)	338 (49.5%)	98 (14.3%)	42 (6.1%)
Energy drinks	217 (31.8%)	216 (31.6%)	120 (17.6%)	93 (13.6%)
Caffeine pills	487 (71.3%)	17 (2.5%)	28 (4.1%)	75 (11.0%)
Cola drinks	257 (37.6%)	227 (33.2%)	107 (15.7%)	52 (7.6%)
Black tea	326 (47.7%)	147 (21.5%)	109 (16.0%)	48 (7.0%)
Green tea	330 (48.3%)	137 (20.1%)	111 (16.3%)	46 (6.7%)

Binary univariable logistic regression analyses showed that none of the independent variables (age, gender, professional status) could predict the use of OTC or illicit and prescription substances, respectively (data not shown). Regarding the sources of information of PN and use of OTC substances for PN, only the category “knowing about it from other students” gained statistical significance (*p* < 0.05).

Regarding the brand names of the energy drinks, the most frequently used energy drink was Red Bull® (*n* = 268, 39.2%) followed by Monster® (*n* = 118, 17.3%), Rockstar® (*n* = 100, 14.6%) and Relentless® (*n* = 68, 10.0%) and further brands that are less well-known (e.g., Bullit Energy®, Magic Man®, Grizzly Energy®, Bizz up Energy®, Black Cat®, etc.).

Age of first use (mean values*, M*, ± standard deviation, *SD*) were for coffee 16.0 ± 2.9 years and for energy drinks 16.7 ± 3.8 years, for caffeine pills 19.1 ± 3.5 years, for cola drinks 10.5 ± 3.3 years, for black tea 14.8 ± 4.9 and for green tea 16.9 ± 5.4 years (see Table 5).

**Table 5:** Age of first use of methylxanthines for neuroenhancement.

**Methylxantine used for neuroenhancement**	**Age of first use**
Coffee	16.0 ± 2.9 years
Energy drinks	16.7 ± 3.8 years
Caffeine pills	19.1 ± 3.5 years
Cola drinks	10.5 ± 3.3 years
Black tea	14.8 ± 4.9 years
Green tea	16.9 ± 5.4 years

**Figure 1:**
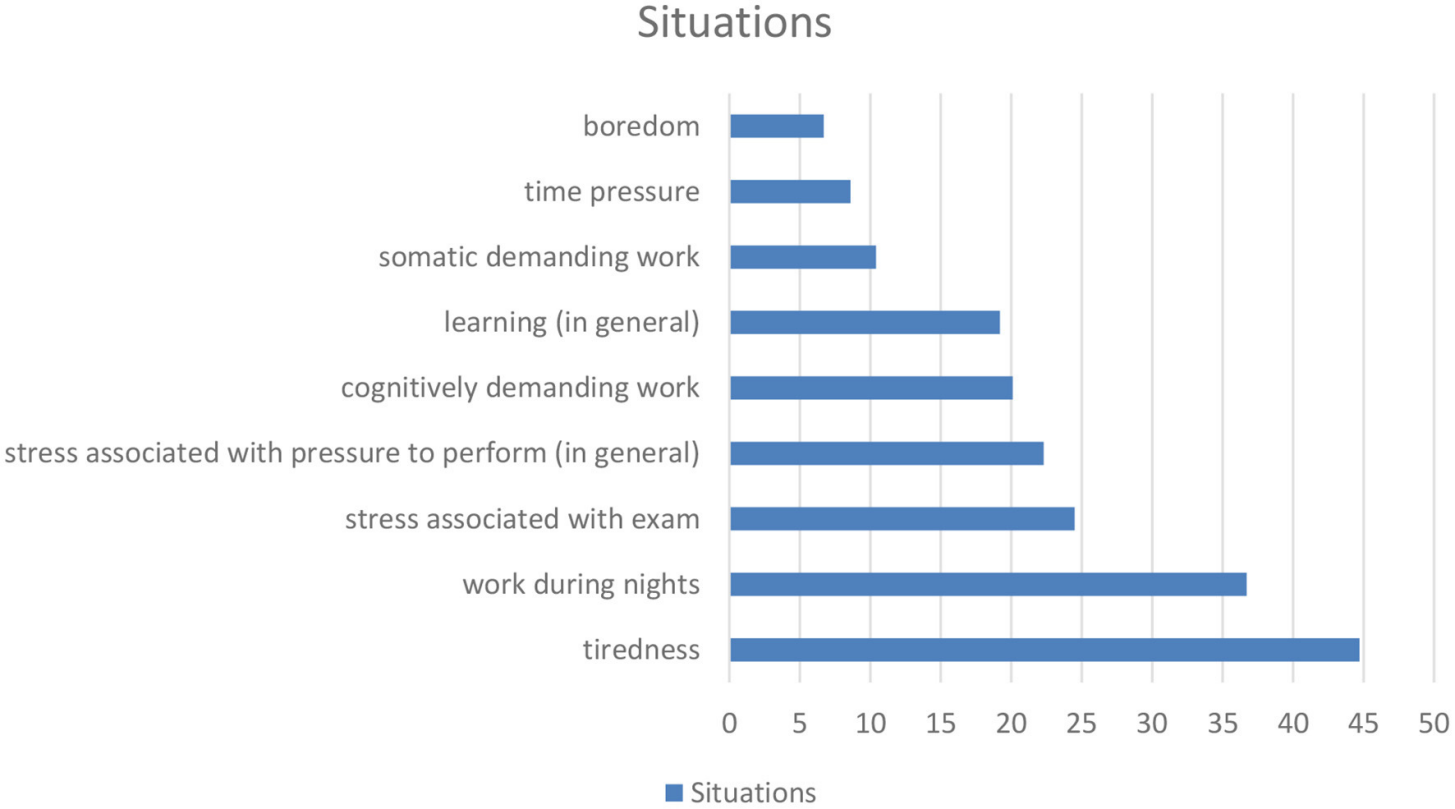
(According to Table 6): Situations of the use of methylxanthines for neuroenhancement.

**Figure 2:**
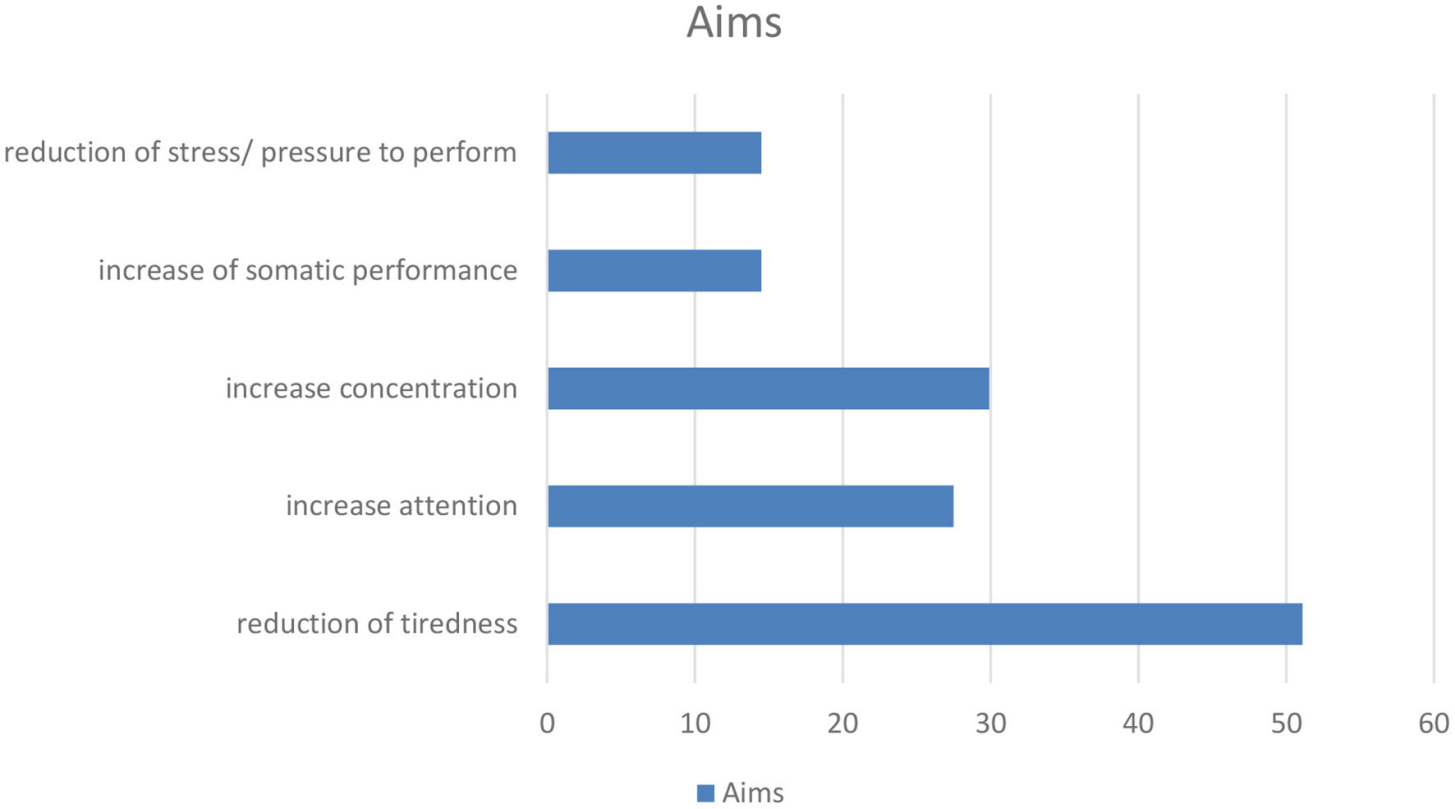
(According to Table 6): Aims for the use of methylxanthines for neuroenhancement.

Asked for specific situations in which methylxanthine containing substances/drinks were used, the most frequently named situation was tiredness (*n* = 305, 44.7%). This aspect was followed by the items work during nights (*n* = 251, 36.7%), examinations and stress associated with examinations (*n* = 167, 24.5%), stress associated with pressure to perform (in general) (*n* = 152, 22.3%), cognitively demanding work (*n* = 137, 20.1%) as well as learning (in general) (*n* = 131, 19.2%), somatic demanding work (*n* = 71, 10.4%), time pressure (*n* = 59, 8.6%), boredom (*n* = 46, 6.7%) and a bad mood (in general) (*n* = 28, 4.1%) (see Figures 1, 2 and Table 6).

**Table 6:** Situations and aims for the use of methylxanthines are used for neuroenhancement.

**Situations**	**Aims**
Tiredness (*n* = 305, 44.7%)	Reduction of tiredness (*n* = 349, 51.1%)
Work during nights (*n* = 251, 36.7%)	Increase attention (*n* = 188, 27.5%)
Stress associated with exam (*n* = 167, 24.5%)	Increase concentration (*n* = 204, 29.9%)
Stress associated with pressure to perform (in general) (*n* = 152, 22.3%)	Increase of somatic performance (*n* = 99, 14.5%)
Cognitively demanding work (*n* = 137, 20.1%)	Reduction of stress/pressure to perform (*n* = 99, 14.5%)
Learning (in general) (*n* = 131, 19.2%)	
Somatic demanding work (*n* = 71, 10.4%)	
Time pressure (*n* = 59, 8.6%)	
Boredom (*n* = 46, 6.7%)	
Bad mood (in general) (*n* = 28, 4.1%)	

According to the above mentioned situations the most frequently stated aim was the reduction of tiredness (*n* = 349, 51.1%). Further frequently stated aims were increase of attention and concentration (attention: *n* = 188, 27.5%; concentration: *n* = 204, 29.9%), increase of somatic performance and reduction of stress/pressure to perform (each *n* = 99, 14.5%) (see Figures 1, 2 and Table 6).

Participants were asked if they had felt an increase regarding somatic and cognitive performance after having used a methylxanthine containing substance/drink: 21.2% (*n* = 145) had felt an increase of somatic performance and 28.4 (*n* = 194) of cognitive performance.

The most common side effects were tachycardia (*n* = 113, 16.5%), pronounced restlessness (*n* = 79, 11.6%), sleeplessness (*n* = 71, 10.4%), jitteriness (*n* = 63, 9.2%) and nervousness (*n* = 65, 9.5%). Infrequent side effects were stomachache (*n* = 34, 5.0%), headache (*n* = 24, 3.5%) as well as nausea and vomiting (*n* = 19, 2.8%) (see Table 7).

**Table 7:** Side effects.

**Side effects**	**Participants**
Tachykardia	16.5% (*n* = 113)
Pronounced restless	11.6% (*n* = 79)
Sleeplessness	10.4% (*n* = 71)
Jitteriness	9.2% (*n* = 63)
Nervousness	9.5% (*n* = 65)
Stomachache	5.0% (*n* = 34)
Headache	3.5% (*n* = 24)
Nausea and vomitus	2.8% (*n* = 19)

Regarding the question, whether participants had mixed energy drinks with alcohol, 9.5% (*n* = 65) stated that they had never done this, 9.4% (*n* = 64) during the last 30 days, 18% during the last year (*n* = 123) and 20.5% (*n* = 140) during a period longer than 1 year ago.

Daily use of coffee varied between one and eight cups (see Table 8): 35.0% (*n* = 239) reported to drink one cup per day, 14.9% (*n* = 102) two cups, 8.2% (*n* = 56) three cups, 3.5% (*n* = 24) four cups, 2.3% (*n* = 16) five cups, 1.3% (*n* = 9) six cups, no one seven cups and 0.3% (*n* = 2) eight cups.

**Table 8:** Daily cups of coffee.

**Cups of coffee per day**	**Participants**
One cup	35.0% (*n* = 239)
Two cups	14.9 (*n* = 102)
Three cups	8.2% (*n* = 56)
Four cups	3.5% (*n* = 24)
Five cups	2.3% (*n* = 16)
Six cups	1.3% (*n* = 9)
Seven cups	/
Eight cups	0.3% (*n* = 2)

## Discussion

The present study focused on a population of students and recent alumni from a German university. During this life phase, exams and first challenges of the new job after graduation might increase the desire to enhance the cognitive performance. However, the decision has to be made whether a legal—which means a methylxanthine containing substance or drink in most cases—or an illegal substance (e.g., amphetamine) should be used for PN. According to higher prevalence rates for caffeinated substances compared to prescription/illicit substances, this study as well as previous studies (8, 21, 22, 26) show, that the majority of subjects tend to the use of a legal substance. This is, in most cases, a methylxanthine containing substance or drink. Therefore, we evaluated the use of “soft” neuroenhancement with caffeinated drinks and substances (caffeine pills) that were used only for the purpose to stimulate cognition (PN). Not all participants were willing to fill out the questionnaire about the use of PN substances and drinks. It could be shown that coffee with a consumption rate of over 70% was the most widely used drink for PN. The next most frequently used substances were energy drinks with a level close to 70%. Taken all caffeinated drinks together, over 88% of all study participants reported to use these drinks for PN.

The current cognitive enhancement debate about academic performance is dominated by the misuse of “prescription drugs” (8). The current study as well as studies from 2011 and 2013 (8, 21) observed low rates for the use of prescription and illicit substances, while others found higher proportions: A French study showed that nearly 70% of medicine/pharmacy students used neuroenhancers within the last 12 month (27). In our study sample the prevalence rates for the use of illegal drugs and prescription drugs (use without medical prescription) was only 1.8%. However, the question for using prescription and/or illicit drugs for PN in the present study was only asked to establish a basis to enable a comparison to other studies. Main focus of this preliminary study was raising data about the use of methylxanthines for PN leading to the problem, that there are nearly no similar studies.

The above mentioned lifetime prevalence of 1.8% for prescription/illicit substances is more or less similar to a previous German study where students showed a life-time prevalence for prescription drugs (e.g., amphetamines, methyphenidate) of 0.8% (last-year and last-month prevalence rates were much lower). For the use of illicit drugs, a life-time prevalence for 2.9% was reported for this student group (22). This previous study as well as the present study are in contrast to a study of Maier et al. (8). In our sample of students and associates of the university of Neubrandenburg, the use of prescription substances like methylphenidate was minimal compared to their results. Reason for these differences might include the rural area of Neubrandenburg with possibly less opportunities of getting inappropriate access to drugs on prescription. Another reason could also be different regulations and laws varying from Switzerland to Germany and participant's average age.

In contrast to prescription substances, caffeinated drinks offer a legal alternative for neuroenhancement and are already widely used for leisure use. The fact that the vast majority of students in this study used coffee, green tea (e.g., Club-Mate®), energy drinks (e.g., Red Bull®) and cola drinks to enhance their cognitive performance was somehow surprising. This also means that only a minority of students does not take any substances for PN.

For the majority of participants in our study, the use of caffeinated led to side effects such as sleep disturbances. Caffeinated beverages have been shown to provoke a dose dependent negative effect on sleep onset, time and quality (28). This is in line with the data of the “Arzneimittelfachinformation” according to the regulatory affairs of the EMA (European Medicine Agency) for caffeine tablets. This is due to the mechanism of action of caffeine being the same in all methylxanthine containing substances/drinks (green and black tea as well as in coffee, energy drinks and caffeine tablets).

In our study, we did not find any significant differences regarding OTC or illicit/prescription substance use between men and women, respectively. Previously it has been shown that male students are more likely to use stimulants to improve cognitive performance than female students (9, 29). Beyond that, we could show that only “knowing the experience” of PN from others was able to predict the own use of these stimulants. This clearly indicates that peer effects do play a strong role in this cohort of students and recent alumni.

Motives of caffeine consumption have been evaluated in detail before: Alertness, mood, social, taste, habit and symptom management were factors identified. The motive “taste” appeared highly important among all types of caffeine users (30). This could mean that the widespread flavor motive is a consequence of the association of the flavor with the negative reinforcing effects of caffeine. However, the aspect of “flavor” or “taste” was not addressed in the questionnaire because of the strong focus on PN.

Regarding the situations in which methylxanthines are used, the present study shows tiredness, work during nights, stress, pressure to perform, somatic and cognitively demanding work, learning and time pressure to be the most prevalent situations. The aims (reduction of tiredness, increase of attention and concentration, increase of performance, reduction of stress) are tightly associated with these situations. This could mean that methylxanthine use may be an “instrument” to cope with the above mentioned situations.

Demanding situations being reasons for PN substance use as well as the above mentioned aims are in line with a previous study about caffeine use among surgeons (31). The study by Franke et al. revealed pressure to perform to be a highly relevant motive for the use of caffeine. These are the same situations in which prescription and illicit stimulants are used for PN among surgeons (32). Beyond that, the situations are quite similar among students regarding the use of caffeine, prescription and illicit substances for PN purposes (8, 21, 22, 26). This leads to the assumption that PN drug use of prescription and illicit substances can be considered as a coping strategy, too (33). Showing similar situations and aims for the use of methylxanthines for PN, the concept of coping strategies seem to be applicable for methylxanthine use (drug and drink) assessed in the present study. PN by prescription/illicit substances seem to have a similar basis as PN by methylxanthine substances/drinks. However, a previous study has shown, that the decision of students to consider prescription/illicit stimulants or caffeine is mainly based on a subjective evaluation of medical and legal (as well as ethical) aspects (17).

Regarding the brand names of the energy drinks, there are no scientific comparable studies. However, Red Bull® seems to be the most widespread and well-known energy drink, at least in our study.

Beyond that, years ago, the question of a risk of dependence caused by the use of methylxanthines has been denied (34). However, in our study energy drinks such as Red Bull® are used together with alcohol. This confirms previous studies showing this habit to be associated with the aspect of “partying” (35, 36). However, out study cannot contribute data to the aspect of using energy drinks with alcohol for partying reasons.

The present study also has some limitations: Firstly, the study took place at just one university and the sample size was relatively small. Furthermore, the group of participants was not completely homogenous (students, alumni, double stressed being students as well as employees). However, the majority of the surveyed students stated to belong to the group of students. Since the survey was spread by social media and other online platforms, anybody could have participated, not only students/alumni of the mentioned university. In summary, our observations cannot be generalized to the whole group of German students. The present study, even if preliminary, is a contribution to the field, adding data and new insights to an underinvestigated field which has to be considered as a strength. Further studies with preferably more representative data need to be conducted in the future. Secondly, mental disorders like depression, insomnia or attention deficit disorder (ADHD) were not taken into consideration even though these disorders might alter the use of neurostimulants. Thirdly, beyond the physically and mentally stimulating effect of methylxanthines, there are two further aspects of methylxanthines use: (a) a flavoring aspect of the taste of caffeine and (b) the question of a cultural habit. Even if the questionnaire stressed the PN aspect, it has to be considered that some participants may have merged the three aspects in their mind which may have led to less exact results.

Finally, there is a high prevalence of using OTC substances such as methylxanthines for PN compared to the use of prescription/illicit substances. Furthermore, there seem to be an enormous pressure to perform among students and alumni. If this pressure persists, a “switch” from the use of OTC substances to prescription/illicit substances may occur according to the so called gate-way hypothesis (the use of legal substances may reduce the obstacle to use prescription/illicit substances) (19).

## Data Availability Statement

The raw data supporting the conclusions of this article will be made available by the authors, without undue reservation.

## Ethics Statement

The studies involving human participants were reviewed and approved by Neubrandenburg, Approval No. BB 045/14. The patients/participants provided their written informed consent to participate in this study.

## Author Contributions

AF was responsible for the study design, development of the questionnaire, and for data acquisition. GK, DK, LP, and AC were responsible for the analysis of the data. The aforementioned authors together with MS, TJ, and OP contributed to the writing process of the manuscript and the process of reading process. All authors contributed to the article and approved the submitted version.

## Conflict of Interest

The authors declare that the research was conducted in the absence of any commercial or financial relationships that could be construed as a potential conflict of interest.
